# Red Wine and Garlic as a Possible Alternative to Minimize the Use of Nitrite for Controlling *Clostridium Sporogenes* and *Salmonella* in a Cured Sausage: Safety and Sensory Implications

**DOI:** 10.3390/foods9020206

**Published:** 2020-02-17

**Authors:** Luis Patarata, Sílvia Martins, José António Silva, Maria João Fraqueza

**Affiliations:** 1 CECAV, Animal and Veterinary Research Center, University of Trás-os-Montes e Alto Douro, 5001-801 Vila Real, Portugal; silviadgmartins@gmail.com (S.M.); jasilva@utad.pt (J.A.S.); 2 CIISA, Centre for Interdisciplinary Research in Animal Health, Faculty of Veterinary Medicine, University of Lisbon, Avenida da Universidade Técnica, Pólo Universitário do Alto da Ajuda, 1300-477 Lisbon, Portugal; mjoaofraqueza@fmv.ulisboa.pt

**Keywords:** dry-cured sausages, nitrite, wine, *Clostridium*, *Salmonella*

## Abstract

The use of nitrite in meat products has been questioned due to its potential association with colon cancer. This work aimed to evaluate the behavior of *Clostridium sporogenes* (used as a surrogate for *Cl. botulinum*) and *Salmonella* in a dry-cured sausage, *chouriço*, made with and without nitrite and nitrate or with red wine and garlic, and to study the sensory implications through a consumer test. The survival of *Cl. sporogenes* and *Salmonella* was determined mainly by the reduction in water activity (a_w_), but the use of wine or wine and garlic contributed to the control of *Salmonella* during processing. The challenge test with *Cl. sporogenes* revealed no effect of the curing salts, wine, or garlic on the population of this microorganism. The use of curing salts resulted in a more reddish color that was recognized by the consumer as over-cured and artificial when compared with *chouriço* made with wine or wine and garlic, which were better rated in the hedonic test. In cured sausages of small caliber, the use of nitrite might be reconsidered, as the values of a_w_ necessary to inhibit *Clostridium* toxinogenesis and growth are achieved rapidly.

## 1. Introduction

Dry sausages are cured fermented meat products, usually made from pork seasoned with salt, herbs, and spices, and they commonly include curing salts—nitrite and/or nitrate [1]. In wine-producing regions, there is a strong association between wine and its use in the preparation of meat products [2,3,4]. The Douro region, in the north of Portugal, has a long tradition of winemaking. Its landscape of vineyards was recognized to be of United Nations Educational, Scientific, and Cultural Organization (UNESCO) World Heritage interest [5]. Linked to more than 2000 years of wine producing, the local gastronomy of the region has taken advantage of the easy access to wine. In the tradition of meat product manufacturing, several products are seasoned with generous amounts of red wine, sometimes as high as 10% (*v/w*), which is added in the batter used for *chouriço*—a filled sausage with a natural casing that is tied in a horseshoe shape and cold smoked and dried. Wine can also be used in a marinade for whole piece products, such as cured pork loins [6,7]. In these meat products, wine has an indubitable sensory effect on their color and aroma and probably exerts an antimicrobial effect due to the presence of organic acids, ethanol, and phenolic compounds [8,9]. Additionally, it is used in combination with garlic, which has known antimicrobial activity due to the sulfur compounds released when crushed [10,11]. Dry sausages have a long history of safe use, and reports on foodborne diseases associated with them are rare [12,13]. Their safety is assured by an ensemble of hurdles that contributes to the inhibition, or eventual death, of foodborne pathogens, commonly known as hurdle technology [1]. These hurdles include, among others, reduced water activity, the activity of competitive microflora, the reduction of pH, smoke, and the antimicrobial effect of ingredients and chemical preservatives. The main chemical additive used as a preservative in dry meat products is nitrite. The risk of *Clostridium botulinum* growth and toxinogenesis justifies the use of nitrite in some dry meat products. Furthermore, the use of nitrite contributes to the color stability of meat products [14]. The inhibition of *Salmonella* is also a concern, as it is one of the most frequent pathogens found in the meat product industry [15].

In 2015, the World Health Organization reported that consumption of meat products may increase the risk of colon cancer, among other aspects, due to the presence of N-nitroso compounds. The risk described involves the consumption of all foods using nitrite and/or nitrate in their manufacture [16,17]. We need to study alternative strategies to the use of nitrite, because there is a great difficulty in finding substitutes to fulfill its purpose. The knowledge available is also limited with respect to safety and sensory characteristics of products in which the additive is suppressed or reduced. The elimination of nitrite without assessing the risk factors involved may result in uncontrolled growth of pathogens of high concern in this industry or in lower acceptability or other undesirable sensory changes, causing economic losses [18,19,20].

The aim of this work was to (1) evaluate the behavior of *Clostridium sporogenes* (used as a surrogate for *Cl. botulinum*) and *Salmonella* in *chouriço* through a challenge test in *chouriço* made with and without nitrite and nitrate or with red wine and garlic and (2) study the sensory consequences and acceptability of the formulations used through a consumer test.

## 2. Materials and Methods

### 2.1. Experimental Design

**Figure 1:**
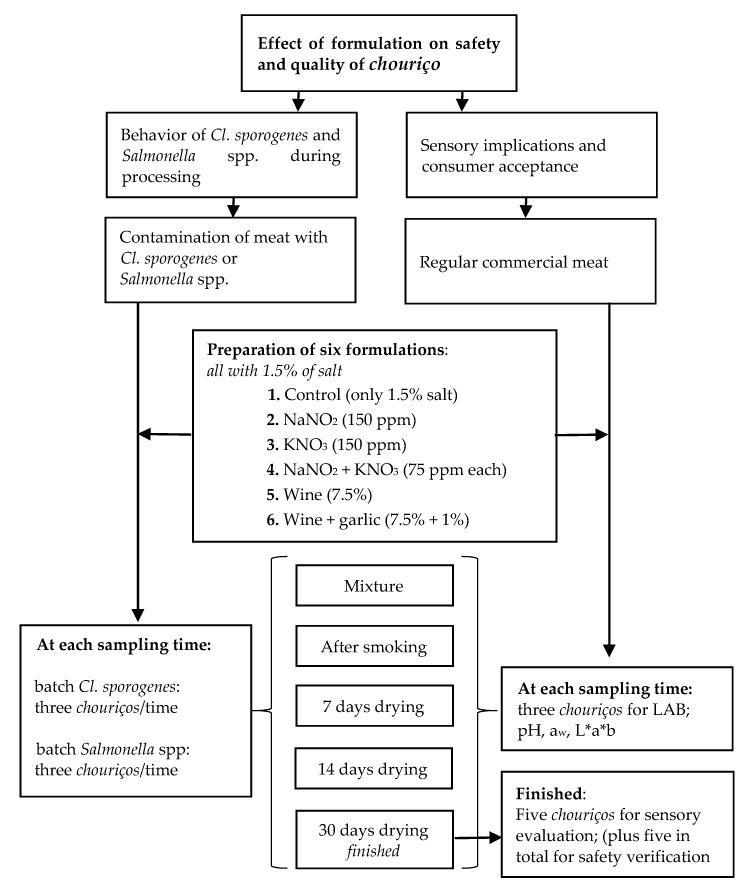
Experimental design.

The design of the experiment is presented in Figure 1. *Chouriços* were prepared with six different formulations: (1) control only with salt, (2) sodium nitrite (150 ppm), (3) potassium nitrate (150 ppm), (4) sodium nitrite plus potassium nitrate (75 ppm each), (5) red wine (7.5%); and (6) red wine plus garlic (7.5% and 1%, respectively). The levels of nitrite and nitrate used alone were the maximum allowed by European law [21]. When combined, we opted to use a total amount for both, avoiding exceeding the limit of 150 mg/kg of each additive. These formulations were used in a first experiment to perform a challenge test evaluating the behavior of *Cl. sporogenes*, used as a surrogate for *Cl. botulinum*, and *Salmonella* inoculated in the meat used for the *chouriço* preparation and in a second experiment to study the influence on the sensory characteristics of the meat product. The samples were taken for analysis after mixture and smoking and at 7, 14, and 30 days of drying, when the product was considered finished. Non-inoculated samples were also used to monitor pH, water activity (a_w_), and lactic acid bacteria (LAB) of natural fermentation. From each sampling step and time, three samples were taken. Color parameters L*a*b* and sensory analysis was undertaken only with the non-inoculated finished product. 

### 2.2. Bacterial Strains and Preparation of Inoculum

*Clostridium sporogenes* (DSM 767) was used as a surrogate for *Cl. Botulinum*, because it was not possible to use that pathogen under the available laboratory conditions. This surrogate shares many common characteristics with *Cl. botulinum*, namely the mechanisms of nitrite inhibition and other parameters used in food preservation [22]. The suspension of *Cl. sporogenes* used for inoculation was prepared from a fresh culture in reinforced clostridium medium (RCM). The third generation of the culture was poured into a Roux flask containing about 150 mL of PA3679 culture medium modified according to [23]. The cultures were incubated for 72h at 30 °C in anaerobiosis (Anaerogen, Oxoid, Hampshire, UK). Culture maturity was determined by phase-contrast microscopy to detect the presence of spores, which were in most of the observed cells. The culture was collected from the Roux flask, centrifuged, and washed twice with a sterile water solution of 0.85% NaCl. The cell suspension was pasteurized at 80°C for 10 min. The number of spores in the suspension was estimated by seeding successive decimal dilutions in modified PA3679 culture medium followed by incubation at 30 °C in anaerobiosis. The spore suspension was kept refrigerated until use. Before inoculation, the spore suspension was diluted to obtain a level of inoculation of 3–4 log CFU/g. Three strains of *Salmonella* were used. One from a culture collection (*Salmonella enterica subsp. enterica* CECT 4155) and two wild strains, one of which was isolated from an industrial cured sausage after smoking and the other from the production environment during manufacturing. Strains maintained at −18 °C were cultured twice in Brain Heart Infusion (BHI, Biokar, Allonne, France) and incubated 18 to 24 h at 37 °C. Cultures for inoculation were grown individually overnight in 30 mL of BHI, harvested by centrifugation, washed twice, and suspended in a sterile solution of NaCl 0.85%. A mixture of the three *Salmonella* strains was prepared to achieve a level of inoculation of about 6–7 log CFU/g. 

### 2.3. Preparation of Chouriço

Experiments for challenging pathogen behavior and for sensory analysis were prepared separately in time. The first experiment prepared was for sensory analysis, and only after the meat products were concluded did the experiment for pathogen behavior begin. 

All the experiments were prepared using meat and fat from pork belly without skin. The meat was ground (15 mm) (Mainca, Barcelona, Spain). The preparation of the six formulations was made according to the indications in Figure 1. Samples without wine (formulations 1 to 4) had a corresponding amount of water added. Commercial red wine was used with a pH of 3.5 and 12% ethanol. After mixing, the batter rested overnight (16 to 18 h). The batter was filled into natural thin casings (40–50 mm diameter) and tied in a horseshoe shape (each sausage weighed ca. 200 g). The *chouriços* were smoked for 3 h in a smoking chamber (Begarat, Thermaxs 100EC, Berlin, Germany) by burning beech wood scraps using electric resistance to produce smoke. The maximum temperature during smoking was never higher than 30 °C. This was followed by drying at 15 °C, 85% Relative Humidity (Aralab Fitoclima, Rio de Mouro, Portugal) for 30 days. Three samples were collected for analysis 4 h after the preparation of the batter, after smoking, and at 7, 14, and 30 days of drying. 

For the challenge test with pathogens, the preparation was similar to that used for the sensory analysis, but the minced meat was contaminated with *Cl. sporogenes* or *Salmonella* in separate batches by adding 10 mL of a suspension of the pathogen to achieve the desired contamination level, 3–4 log CFU/g for *Cl. sporogenes* and 6–7 log CFU/g for *Salmonella.*

### 2.4. Bacterial Enumeration

For the samples inoculated with *Salmonella,* an initial dilution of 1:10 was prepared in NaCl 0.85% solution. A serial decimal dilution was prepared and spread on Hektoen enteric agar (Biokar, France). After incubation at 37 °C for 24 h, characteristic colonies were counted. From each countable petri dish, five colonies were subcultured in COMPASS *Salmonella* Agar (Biokar, Allonne France) to check their identity, and the counts were corrected proportionally. When low counts were expected, the initial dilution was 1:5, and an inoculation of 0.5 mL of the first dilution was spread in two Petri dishes (0.25 each), allowed to dry in the laminar flow chamber to avoid biofilm formation, and counted as the total colonies in both dishes. The samples inoculated with *Cl. sporogenes* were diluted at 1:5 in NaCl 0.85% solution, pasteurized at 80 °C for 10 min, and incorporated in modified PA3679. Decimal dilutions were prepared when necessary. The incubation was made at 30 °C in anaerobiosis during 72 h. Lactic acid bacteria were enumerated in Man Rogosa Sharpe (MRS, Biokar, Allonne, France). The results were presented as log CFU/g. For statistical purposes, when the microorganism count was below the detection limit, it was considered to be zero. Estimated count was considered for data analysis when countable colonies were present but below the countable range. In the results, there are mean values below the unit, due to the mean of the three repetitions including null values in some instances.

### 2.5. PH and Water Activity

The pH of the cured sausage was measured directly using a pH meter (model MicropH 2002, Crison, Barcelona, Spain). Water activity was measured in a Rotronic Hygroscope DT apparatus with a WA40 probe (Rotronic, Bassersdorf, Switzerland).

### 2.6. Nitrite and Nitrate

Nitrite and nitrate contents were determined following ISO methods. The spectrometric determination at 540 nm was made after nitrite reaction with sulfanilamide and *N*-1-naphthylethylenediamine. Nitrate was reduced to nitrite using cadmium, and the amount of nitrate was determined via the difference between total nitrite after reduction and nitrite originally present [24,25].

### 2.7. Instrumental Color Measurement

The color was measured with a tristimulus color analyzer Minolta CR 310 (Minolta, Osaka, Japan) with a standard illuminant D_65_, using L*a*b* color space. The area measured was a 50 mm diameter in the homogenate of the previously minced samples compacted in a 90 mm petri dish.

### 2.8. Sensory Evaluation

To study the influence of the *chouriço* formulation on the sensory characteristics, two approaches were used: an exploratory qualitative research using the focus group (FG) interview methodology to find the main trends and the more adequate vocabulary approach using a check-all-that-apply (CATA) system.

*Focus groups*. The objective was to have a preliminary insight on the main trends of the sensory characteristics and consumer evaluation of the *chouriços* with different formulations. The research was made with three FGs, all composed of consumers with no formal experience in sensory analysis and most of them (> 95%) regular consumers of *chouriço*. The groups were composed of 10 (4 women; 20 to 25 years old), 8 (3 women; 47 to 62 years old), and 7 (3 women; 41 to 55 years old) participants, respectively. The FG interviews were performed as described in [26]. Samples from the six formulations were used in each session in a random order of presentation, different in each of the three FGs. The procedure and the objectives of the FG were explained, and the identification of all participants was recorded. The moderator asked participants to describe the appearance of the *chouriço*, with particular attention to color, aroma, and flavor. Hedonic and affective ratings were also encouraged to be given and recorded. In the closing moment of the FG, the moderator reviewed with participants the main findings of the discussion. The sessions were audio-recorded, and the content was analyzed for the detection of sensory trends for each formulation and to select the vocabulary to use in the CATA test.

*Check-all-that-apply test.* The test was performed with 82 consumers (64.6% women) recruited from the personal and professional contacts of the authors. Consumer ages ranged between 18 and 77 years old (38.0 ± 13.6). Most consumers (87.8%) identified themselves as regular consumers of *chouriço*. Education and occupation were diversified. Students comprised less than 20% of the group. The six samples, each composed of two *chouriço* slices, were presented to each consumer and identified with a random two-letter code. The test was performed as described in [27,28]. Consumers were asked to select from (via checkmark) the presented list of attributes those which applied to each sample. The list of attributes, built based on the results of the FGs, included reddish color, pinkish color, cured color, brownish color, bright color, dull color, red wine color, pale color, dark red color, greenish color, sui generis aroma, and cured aroma. Checkboxes were also included to evaluate consumption and purchasing intention.

*Hedonic evaluation***.** In addition to the checkboxes, the test included a 9-point hedonic scale, to be answered before the CATA attributes were selected. The scale was structured from 1 “dislike extremely” to 9 “like extremely” [29].

### 2.9. Data Analysis

*Clostridium sporogenes* and *Salmonella* counts, L*a*b*, and hedonic evaluation by consumers were analyzed by one-way ANOVA comparing formulations at each sampling time separately. The Tukey test was used to determine the significant differences (*p* < 0.05) between group means. The analysis of the CATA results was done by factor analysis. The frequencies for each attribute between formulations were compared by Cochran test. The mean impact of each CATA attribute in the hedonic evaluation was computed. The frequency of the consumption and purchasing intention for each *chouriço* formulation was compared by a Chi-square test. All the statistics were calculated with XLStat (Addinsoft, Paris, France). Raw data might be accessed at Supplementary Materials.

## 3. Results and discussion

### 3.1. Challenge Test with Cl. Sporogenes and Salmonella

**Figure 2:**
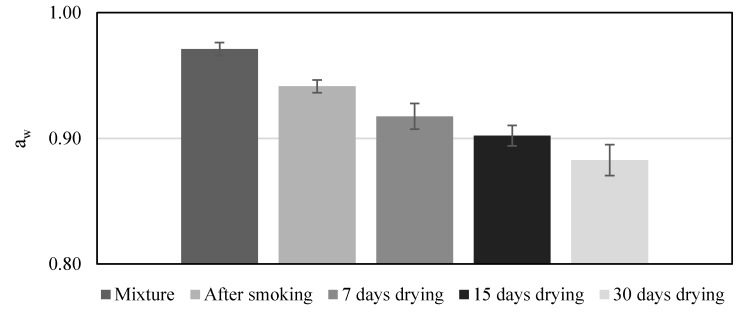
Water activity (mean of all formulations and standard deviation) of the *chouriços* during processing (n = 18).

The counts of *Cl. sporogenes* and *Salmonella* during the processing in the *chouriços* made with the six formulations are shown in Table 1. After the mixture was prepared, the counts were made only for the control samples, as an immediate effect of the formulations on the microorganisms still in the adaptation phase was not expected. The contamination used with *Cl. sporogenes* was at a low level (3.52 ± 0.16 log CFU/g), as the objective was challenging this bacterium in order to evaluate its growth potential. Therefore, the initial contamination was kept as reduced as possible to allow for the counting of survivors during processing and to understand its trend of survival or growth. The challenge test with *Salmonella* was performed with a high level of inoculation, nearly 7 log CFU/g to have the possibility to demonstrate a 5 log reduction during processing, which is commonly used as a performance standard for dry, fermented, and salt-cured products [30]. *Clostridium sporogenes* showed a continuous reduction during the processing of *chouriço*. It went below the detection limit after 15 days of drying when the cured sausage presented an a_w_ of 0.91 (Figure 2). Generally, *Cl. botulinum* growth is inhibited at a_w_ < 0.97 (Group II) or < 0.94 (Group I) [31]. Information on specific a_w_ inhibition values for *Cl. sporogenes* is scarce but, due to the similarities between these two species, it is expected that this species could have a similar sensitivity to low a_w_. The counts of this surrogate for *Cl. botulinum* were always slightly higher in the control samples but without statistical importance (*p* > 0.05). Notwithstanding the reputed inhibitory effect of nitrite on *Clostridium* growth [32], in the present work, it was not observed. In another study with *Cl. sporogenes* [22], the growth of this microorganism was observed when reduced amounts of nitrite (100 mg/kg) were used if any other preservative was not added to the sausages. However, the referenced experiment was made with fresh sausages during storage and without any reduction in a_w_. Even considering the potential microbial inhibitory effect of wine due to its low pH or eventually of garlic due to allicin and other sulfur compounds, the behavior of the bacteria on the control *chouriço* made only with salt indicates that the reduction of a_w_ is the main hurdle to bacteria growth and, as observed in the present work, to the survival of *Cl. sporogenes*. Our results are in accordance with those obtained with Spanish dry fermented sausages inoculated with *Cl. botulinum* [18]. We observed no bacteria growth or toxin production due to the adverse conditions that dry fermented sausages represent for the pathogen, namely the pH, competitive microflora, and a_w_.

*Salmonella* was also strongly influenced by the reduction of a_w_, as it had a reduction of 5 to 6 log CFU/g during sausage processing formulated with curing salts or with wine and wine and garlic. In the control, the absolute reduction was slightly lower but still important, around 4 log CFU/g. After smoking, it was observed that the counts of *Salmonella* in the control *chouriço* were significantly higher than in any other formulation, revealing that both curing salts and wine have an inhibitory effect on this pathogen. This fact, observed particularly in this stage, is justified, because a_w_ is still very favorable to the multiplication of *Salmonella* [33]. It is worthy of note that both sausages formulated with wine presented lower (*p* < 0.05) counts than those prepared with curing salts. The inhibitory effect of wine on *Salmonella* has been demonstrated in culture media and in some meat preparations [9,34]. Although nitrite has been used in meat products mainly to control *Cl. botulinum*, it has an important inhibitory effect against *Salmonella*, which is particularly important if the product pH is higher than 5.2 [15,35]. In the present work, the values of pH were higher than that limit, and *Salmonella* was also inhibited but to a lesser extent in the absence of curing salts or wine. After seven days of drying, the effect of the a_w_ reduction seemed to begin to overlap the effect of different sausage formulations, as the differences between them became narrower but were still present between the control and the *chouriço* made with nitrate, the *chouriço* made with the combination of nitrite and nitrate, and the *chouriço* made with wine. The *Salmonella* counts observed in *chouriço* with nitrite were lower than those in the control but still not significant (*p* ≥ 0.05). From day 7 of drying to day 15, *Salmonella* experienced a reduction of about 2 log CFU/g, maintaining the same pattern of differences. At this stage, the *chouriço* had already reached an a_w_ of 0.91, which might be considered ready to consume [1]. In samples made with wine, a reduction of 5 log in *Salmonella* counts, used as performance criteria for dry-cured or fermented meat products, had already been achieved [30]. The final drying until day 30 resulted in a *chouriço* with an a_w_ between 0.87 and 0.89. Counts were below the detection limit in most of the repetitions. On average, counts were reduced by almost 2 log CFU/g from day 15 of drying. The control *chouriço* after 30 days of drying presented higher (*p* < 0.05) counts of *Salmonella* than cured sausages prepared with nitrite, alone or in combination with nitrate, and than those prepared with wine.

The behavior of the two microorganisms studied in the present work demonstrates that the traditional process of manufacturing cured sausages is safe. Considering the high prevalence of *Salmonella* in the pork [36], the reduction observed due to the drying and the additional reduction achieved with the use of wine raise very interesting perspectives on the validation of microbiological hazard control in meat products made with wine, which are common worldwide in wine-producing regions [3,4,37].

### 3.2. Evolution of pH, Lactic Acid Bacteria, and Water Activity in Chouriços with Different Formulations 

**Figure 3:**
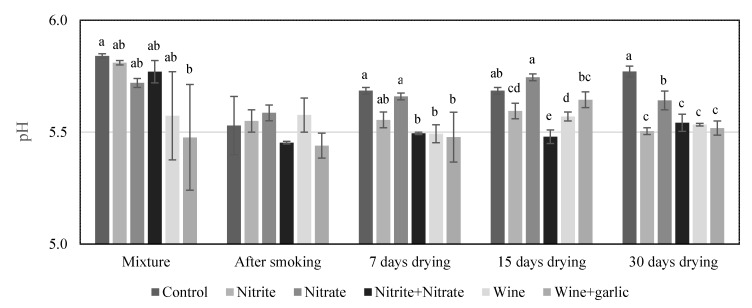
pH evolution (mean and standard deviation) during the processing of the *chouriços* prepared with different formulations (n = 3); **a,b** Bars in the same sampling time with different letters are statistically different (*p* < 0.05).

**Figure 4:**
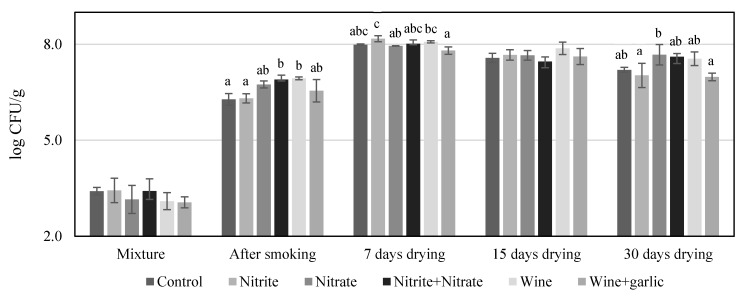
Counts of lactic acid bacteria (mean and standard deviation) during the processing of the *chouriços* prepared with different formulations (n = 3); **a,b** Bars in the same sampling time with different letters are statistically different (*p* < 0.05).

The pH evolution during the processing of the *chouriços* with different formulations was between 5.4 and 5.8 (Figure 3). In the *chouriço* made with wine, the initial pH was lower in 0.2–0.3 units than the pH of those made without wine. The low pH of the wine, around 3.5, added to the meat resulted in acidification of the mixture but with considerable differences between repetitions, as observed by the higher dispersions around the mean values. In the control *chouriço* and in those with nitrite and/or nitrate, the pH decreased after the smoking step, mainly due to the shift in the LAB growth (Figure 4), and increased again during drying. This is commonly observed in this type of meat products, because the meat needs time to absorb the acids and manifest its buffer capacity and also due to the formation of basic compounds associated with the catabolism of amino acids. Punctual differences were observed throughout processing, mainly between the control, which displayed higher values, and the *chouriço* with wine, which displayed lower values. In the final product, the differences between the control and the other formulations was clear, although still small in absolute values.

The counts of LAB were around 3 log CFU/g after mixing. In the smoking stage, associated with heating to 25–30 °C, these microorganisms multiplied to more than 6 log CFU/g. It was on day 7 of drying that LAB achieved the maximum count, around 8 log CFU/g. The increase in the population of LAB is expected in *chouriço*, which, even when not inoculated with a starter culture, shows a dominance of the microbiota by these fermentative microorganisms [13]. Lactic acid bacteria probably contributed to the behavior observed with *Cl. sporogenes* and *Salmonella*. Even considering that the differences in pH were residual, as no fermentable sugars are used for producing *chouriço*, LAB might have contributed to the control of the challenged microorganisms through competition or other inhibitory mechanisms [38]. During processing, the differences observed were punctual and had no apparent relationship with the formulation. 

The a_w_ (Figure 2) was similar (*p* ≥ 0.05) between the formulations at each sampling time. The results presented in Figure 4 are the mean of all the formulations. A decrease from an initial mean value of 0.97 to a mean final value of 0.88 was observed. After 15 days of drying, the a_w_ was sufficiently reduced to consider the *chouriço* finished and ready for consumption, as it was lower than 0.91 [39]. 

### 3.3. Nitrite and Nitrate

The levels of nitrite and nitrate were residual in the control *chouriço* and in those made with wine and wine and garlic. In the cured sausages formulated with these additives, the levels were also very reduced, less than 10 ppm of nitrite and less than 15 ppm for nitrate. *Chouriços* that did not use these additives showed residual values. The *chouriço* with the nitrite and nitrate pair showed the highest concentration of nitrite at 5 mg/kg and residual nitrate at 15 mg/kg. The nitrite and nitrate content was reduced to about 10% of the added amount. These levels of nitrite in the finished product are due to its reduction to nitric oxide, which in turn reacts with the hemic group of the myoglobin and with other components of the sausage, resulting in a very small concentration in the finished product [19]. Residual nitrite at the end of the manufacturing process depends on several factors supporting the reduction of nitrite to nitric oxide, such as the pH of the medium, time and temperature of processing, microbial load present in the raw material, and addition of antioxidants [40]. The residual levels of nitrite and nitrate observed in the present work were in the same range of those usually observed in commercial products [41].

### 3.4. Color Parameters L*a*b*

The evaluation of the color parameters between the six formulations is presented in Table 2. L* values ranged from 43.36 ± 0.40 in the control *chouriço* to 46.79 ± 1.04 in the *chouriço* prepared with nitrite plus nitrate. The only statistical difference observed was between these two extremes. The redness of the *chouriços* evaluated by the a* value presented considerable differences between formulations. The *chouriço* prepared with nitrite was redder (a* 17.59 ± 0.51) than any other studied formulation. Samples with nitrate or a combination of both nitrite and nitrate were also redder than the control. Although red wine gives a characteristic color to the *chouriço*, the a* values were lower than those observed in the meat product with curing salts. The color parameters L* and a* observed in the present work in the *chouriço* with curing salts were similar to those observed in Spanish dry-cured sausages made with nitrite, which is associated with the nitrosation of the myoglobin to nitrosomyoglobin [42]. The color parameter b*, measuring yellowness in the positive range, was higher than those referred to in the previous reference and in Pamplona *chouriços* [43]. Due to the heterogeneous nature of the *chouriço*, in the present work, the color was measured using homogenized samples. The dispersion of fat in the mixture might have increased the yellow color of the *chouriço*, because fat has a more intense yellow color, particularly due to oxidation [44]. The *chouriço* prepared with wine and garlic presented a particularly high b* component, suggesting that garlic has an interaction with wine pigments modifying its color. The color of red wine is due mainly to anthocyanins and other phenolic compounds. Anthocyanins are prone to oxidation, resulting in the change of color from red to yellow/brown [45] with higher b* values [46]. Garlic is usually considered to have antioxidant capabilities; however, there is strong evidence that it can also be highly pro-oxidant, depending on the substrate in use and the experimental conditions [47,48]. Taking that into consideration, garlic might have an oxidative effect on wine pigments, turning them browner with higher b* values.

### 3.5. Sensory Implications of Different Chouriço Formulations 

To evaluate the sensory implications of the different *chouriço* formulations, three focus groups were carried out to evaluate the appearance, aroma, and flavor of the meat cured sausage (Table 3). Focus group methodology has been used as an exploratory technique to evaluate sensory characteristics and consumer trends [49,50,51] and to generate vocabulary for further sensory analysis [52,53]. The control *chouriço* was identified as having a pale color and a short flavor, as was expected because it was prepared only with salt. The *chouriço* prepared with nitrite, nitrate, or the combination of both had an “over-cured” appearance, with a color recognized by participants as artificial, and an aroma not recognized as *chouriço* but, rather, as bacon or cured ham; this resulted from the lack of aromatization with wine and spices usually present in this product. Both the *chouriços* made with wine or wine and garlic presented a dark pink or dark red color, recognized by participants as the characteristic color resulting from the use of red wine. Consumers associated the appearance of these *chouriços* with those that are homemade. The aroma was considered characteristic, with detectable notes of wine and garlic, when present. That aroma was associated with the characteristic of *chouriço* and was generally well rated by participants in the FGs. 

**Figure 5:**
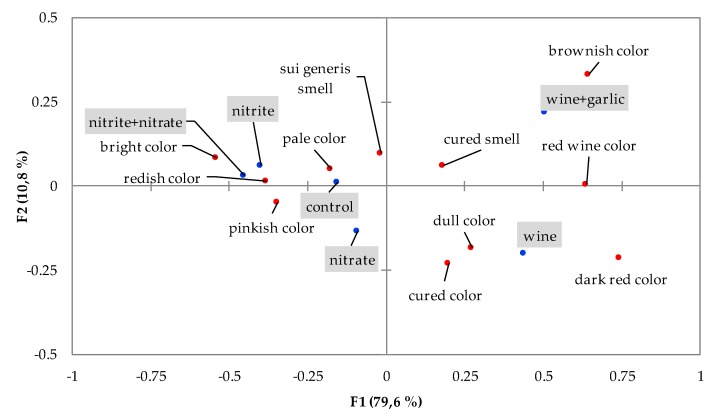
Attributes of the *chouriços* with different formulations tested (grey boxes) in the space defined by the first two factors.

Cured sausages with different formulations were tested by 82 consumers using a CATA test. They identified the attributes of *chouriço* from a list that was presented to them. The list was constructed based on the results of the focus groups. The identification of the attributes in each formulation was analyzed by multifactorial correspondence analysis. The proportion of consumers who identified each attribute in the *chouriços* with different formulations and the statistical differences assessed by the Cochran test are presented in Table 4. The projection of the first two factors is presented in Figure 5. One attribute given in the list as a possible choice, greenish color, was not used in the analysis, as it had only a residual utilization, with no pattern between formulations. Eight out of the nine aspect attributes considered in the analysis displayed differences between formulations. Reddish color was checked by consumers at a higher rate in samples with nitrite or with nitrite and nitrate. These reddish and pinkish color notes are the result of the use of curing salt. The formation of nitrosomyoglobin, from the bonding of nitric oxide to myoglobin iron, results in more vivid colors in the product [54,55]. The control *chouriço* and those with wine were also considered reddish. Pinkish color was noted by about half of participants in the four formulations without wine. That color had the same frequency of checking by consumers with respect to the control samples and those prepared with nitrite and/or nitrate. Cured color was attributed mainly to the *chouriço* made with wine and wine plus garlic. The red wine color that characterizes these samples was considered by consumers to be the cured color, more than the pink and bright color achieved in the samples with nitrite salts. The familiarity of consumers with the characteristic red wine color drove them to consider *chouriços* using red wine as having the cured color. Consumer preference is usually positively influenced by familiarity with the product’s appearance and a better rating of color, as demonstrated in [56]. The *chouriço* prepared with wine and garlic had a high frequency (45%) of references to the brownish color, clearly different from the samples prepared only with wine, indicating that garlic influences the color of the product, through the modification of wine, meat pigments, or both. 

When the results of the CATA test were compared with those of the L*a*b* parameters, they were coherent, with the reddish or pink colors associated with the samples made with curing salts and the less intense red colors associated with the wine formulations. The brownish color observed in the *chouriço* made without nitrite might be related to the oxidation of heme pigments to metmyoglobin, resulting in the formation of a dull brownish color [57]. However, in this work, that mechanism was probably mediated by wine components, because the control *chouriço* was not recognized as being as brown as those prepared with wine. The high b* values observed in the *chouriço* with wine and garlic were recognized by consumers as having a brownish color. The eventual pro-oxidant effect of the garlic components on the wine pigments was previously discussed above. In other studies, in meat products involving the addition of garlic but not wine, a higher yellowness was also observed, which was attributed to the natural yellow color of garlic [58]. Considering the level of addition of 1% of fresh garlic we used, it was probably not enough to produce, by itself, significant changes in color. Another factor that might explain the color variations in the *chouriço* made with wine and garlic might be associated with myoglobin. If, due to the potential pro-oxidative effect of garlic sulfur compounds, the heme group of myoglobin is oxidized from the ferrous to the ferric form, this results in a higher proportion of metmyoglobin and an increase in the brown color of the meat [55]. 

The sui generis aroma of the *chouriços* was similar between the six formulations, but the cured aroma followed the same trend as the cured color, with a higher frequency in samples made with wine. It is expected that the use of nitrite influences the aroma of cured sausages, but it does not necessarily have an influence on consumer choice, as observed in Italian cured sausages [59,60]. The cured aroma consumers detected in the present work is probably linked to the characteristic *chouriço* aroma that is familiar to them, with wine and garlic notes. In previous studies [61], with a group of consumers similar to the one used in the present work, it was observed that the use of garlic essential oil was, among several essential oils, the only one accepted by consumers, which was attributed to their familiarity with that aroma. 

The overall relationship between the attributes and between attributes and formulations can be observed in Figure 5. The projection of the first two factors accounts for 90.4% of the explained information. In Figure 5, it is possible to observe the clear discrimination between the samples with and without wine, with the attributes related to the specific color given by the red wine associated with cured attributes, both for color and for aroma. The use of nitrite and/or nitrate was associated with more bright colors but not with the recognized characteristic cured color. 

**Figure 6:**
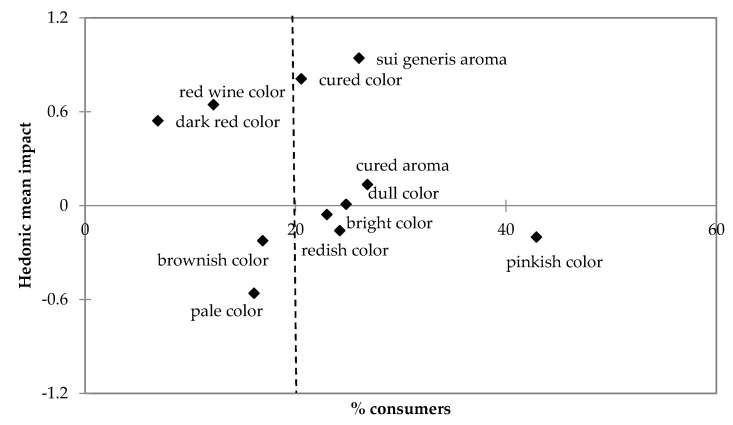
Mean impact of the attributes checked by consumers on the hedonic evaluation. The dashed vertical line defines 20% of consumers.

When the impact of the attributes on the hedonic evaluation was tested (Figure 6), it was observed that the cured color and sui generis aroma were those with a higher positive impact on the hedonic mean, nearly one unit in the 9-point scale. Red wine color and dark red color also had a positive impact, but as these attributes were checked by less than 20% of consumers, at the left of the dashed line in the figure, these values have a limited interest. Color notes associated with the use of nitrite salts, bright reddish and pinkish colors, had a negative impact, still small in value, on the hedonic evaluation of the *chouriço*. That trend was latent in the speech of the focus groups and was probably linked to lower approval scores, as is commonly found when consumers have knowledge of the presence of chemical additives in foods [56,62]. The ANOVA comparison of hedonic evaluation between the six formulations is presented in Table 5. In the same way as with the finding of the hedonic mean impact of the CATA attributes, the only situations detected as statistically different were the *chouriço* made with wine and garlic, with the highest evaluation (5.95 ± 1.47), and the *chouriço* made with nitrite, with the lowest (5.22 ± 1.70). The other four formulations were not statistically different from each other. In Table 5, it is also possible to observe the proportion of consumers indicating the intention to consume or to purchase the products with each formulation. No differences were observed in the consumption intention. In the purchasing intention, the trend was the same as observed for the hedonic evaluation, with the *chouriço* made with wine and garlic having the highest preference scores and that made with nitrite with the lowest. The preference of consumers for *chouriço* without nitrite or nitrate might be related to the more characteristic aromatization of those including wine or wine plus garlic but may also be due to the fact that they recognize these products as “less industrial” or more similar to homemade products, which are recognized as healthier due to the absence of food additives, as was observed from analyzing comments during the FGs and the trends found with the CATA and hedonic evaluations. Some groups of consumers have a serious concern about the healthiness of foods with chemical additives, and they are willing to pay more for “green-label” foods [62,63]. Although in the present work, the use of additives was not revealed to consumers, the appearance of the products, namely their bright red color, was suggestive of the use of additives, which might have impaired the appreciation for such products. These findings suggest that more than the sensory characteristics of the product, it is very important to consider the perception that a consumer has of its safety and healthiness, as well as the habits of consumers [64,65].

## 4. Conclusions

The use of red wine and red wine combined with garlic was shown to be an interesting alternative to control biological hazards in the manufacturing of *chouriço*. The survival of *Cl. sporogenes* and *Salmonella* was determined mainly by the reduction in a_w_, but the use of wine or wine and garlic was revealed to increase the destruction of *Salmonella* during processing. The challenge test with *Cl. sporogenes*, used as a surrogate for *Cl. botulinum*, showed no effect of the use of curing salts, wine, or garlic. In cured sausages of small size, such as *chouriço*, the use of curing salts to control *Clostridium* might be reconsidered, as the values of a_w_ necessary to inhibit toxin genesis and growth are achieved rapidly. From the sensory point of view, the use of curing salts results in a more reddish color, but it is not recognized by consumers as a sensory advantage. The cured color obtained with curing salts was recognized as over-cured and artificial when compared with *chouriço* made with wine or wine and garlic, which was better liked. The unhealthy reputation of meat products is due to several factors, namely high fat and salt amounts and the eventual presence of potentially carcinogenic compounds and biological hazards. Returning to a simpler manufacturing process, based on the adequate control of drying and the additional inhibitory effect of wine, without using nitrite, will result in safe and sensorially adequate products, while reducing the carcinogenic risk inherent to cured meat products.

This study had an inherent limitation—the use of a surrogate for *Cl. botulinum* and not the pathogen itself. The consumers test was made with *chouriço* prepared with curing salts but without wine and garlic, which introduced a potential bias, because only the *chouriços* without curing salts had the traditional seasoning that consumers recognize as typical.

## Figures and Tables

**Table 1:** Counts (log CFU/g) of *Cl. sporogenes* and *Salmonella* in *chouriços* prepared with different formulations during processing (n = 3).

Microorganisms Processing Phase	Control	Nitrite	Nitrate	Nitrite + Nitrate	Wine	Wine + Garlic	*p*
*Cl. sporogenes*							
Mixture	3.52 ± 0.16 ^Ψ^						
After smoking	2.13 ± 0.21	1.62 ± 0.28	1.84 ± 0.06	1.65 ± 0.16	1.70 ± 0.66	1.72 ± 0.12	0.418
7 days drying	1.66 ± 0.31	1.36 ± 0.44	1.68 ± 0.03	1.38 ± 0.44	1.86 ± 0.20	1.81 ± 0.23	0.294
15 days drying	0.31 ± 0.12	<LD	<LD	<LD	<LD	<LD	0.152
30 days drying	0.19 ± 0.32	<LD	<LD	<LD	<LD	<LD	0.416
*Salmonella*							
Mixture	6.79 ± 0.41 ^Ψ^						
After smoking	8.04 ± 0.17a	7.12 ± 0.05b	6.70 ± 0.04b	7.04 ± 0.19b	6.18 ± 0.16c	5.78 ± 0.23c	<0.001
7 days drying	5.36 ± 0.49a	4.71 ± 0.19ab	4.19 ± 0.19b	4.33 ± 0.57b	3.99 ± 0.09b	3.91 ± 0.19b	0.002
15 days drying	3.44 ± 0.44a	2.71 ± 0.19ab	2.19 ± 0.19b	2.33 ± 0.57b	1.96 ± 0.10b	1.85 ± 0.35b	0.001
30 days drying	2.39 ± 0.21a	0.63 ± 0.55b	0.77 ± 0.68ab	0.49 ± 0.85b	0.33 ± 0.58b	0.33 ± 0.58b	0.010

^Ψ^ Counts were made only in control samples, once an immediate effect of the formulations on the microorganism was not expected. a,b, Means in the same row followed by different letters are statistically different (*p* < 0.05).

**Table 2:** Color parameters in the *chouriços* prepared with different formulations after 30 days of drying (n = 3).

Color Parameter	Control	Nitrite	Nitrate	Nitrite + Nitrate	Wine	Wine + Garlic	*p*
L*	43.36 ± 0.40b	45.04 ± 0.11ab	44.12 ± 0.96ab	46.79 ± 1.04a	45.75 ± 1.27ab	45.59 ± 1.21ab	0.037
a*	11.94 ± 0.61e	17.59 ± 0.51a	15.92 ± 0.56b	14.87 ± 0.35bc	13.08 ± 0.34de	13.74 ± 0.30cd	<0.001
b*	18.55 ± 0.22c	19.71 ± 0.26bc	19.53 ± 0.85bc	20.31 ± 0.36b	18.29 ± 0.35c	24.04 ± 0.35a	<0.001

a,b, Means in the same row followed by different letters are statistically different (*p* < 0.05).

**Table 3:** Sensory characteristic extracted from the discussion of three focus groups that tasted the *chouriços* prepared with different formulations.

Ingredients	Aspect	Aroma and flavor
Control	Pinky color; bright; pale color	Smoky; short flavor
Nitrite	“Over-cured”; artificial color; looks like cured pork loin	Short aroma and flavor; tastes like bacon
Nitrate	Very pale color	Does not smell like *chouriço*; short aroma and flavor; smell like cured ham
Nitrite + Nitrate	Brighter than the other; color slightly artificial; color seems more like that of cooked ham than *chouriço*; it seems less homemade (than the previous tested); pinkish color	Short aroma; resembles bacon; slightly smoky
Wine	Dark pink color; drier; slightly brownish	Cured aroma; characteristic wine aroma (intense)
Wine + Garlic	Dark red; homemade quality; red wine color; moister	Characteristic aroma to wine and garlic; tasty; good flavor

**Table 4:** Proportion of consumers identifying each attribute in the *chouriços* with different formulations.

Attributes	Control	Nitrite	Nitrate	Nitrite + Nitrate	Wine	Wine + Garlic	*p*
Reddish color	0.21ab	0.37b	0.17a	0.38b	0.20ab	0.13a	<0.001
Pinkish color	0.50b	0.54b	0.51b	0.52b	0.27a	0.23a	<0.001
Cured color	0.12a	0.17a	0.20a	0.16a	0.38b	0.21ab	0.000
Brownish color	0.10ab	0.07ab	0.10ab	0.06a	0.23b	0.45c	<0.001
Bright color	0.256ab	0.39b	0.17a	0.37b	0.10a	0.10a	<0.001
Dull color	0.22abc	0.11a	0.29abc	0.16ab	0.40c	0.31bc	<0.001
Red wine color	0.07a	0.07a	0.06a	0.02a	0.26b	0.24b	<0.001
Pale color	0.20a	0.13a	0.18a	0.20a	0.10a	0.16a	0.353
Dark red color	0.06abc	0.01ab	0.05ab	0a	0.17c	0.12bc	<0.001
Sui generis aroma	0.26a	0.27a	0.23a	0.24a	0.23a	0.33a	0.536
Cured aroma	0.29ab	0.21a	0.21a	0.18a	0.33ab	0.39b	0.002

**a,b**, Means in the same row followed by different letters are statistically different (*p* <0.05).

**Table 5:** Hedonic evaluation, consumption and purchasing intention with respect to the *chouriços* prepared with different formulations.

Consumer Evaluations	Control	Nitrite	Nitrate	Nitrite + Nitrate	Wine	Wine + Garlic	*p*
Hedonic ^1^	5.32 ± 1.64ab ^3^	5.22 ± 1.70b	5.46 ± 1.48ab	5.67 ± 1.37ab	5.88 ± 1.53ab	5.95 ± 1.57a	0.011
Consumption ^2^	32.9	34.1	31.7	41.5	39.0	39.0	0.733
Purchasing ^3^	20.7 (−0.20)	13.4 (−1.96)	14.6 (−1.37)	24.4 (0.69)	20.7 (−0.20)	35.4 (3.35) ^4^	0.009

^1^ Results expressed in mean ± standard deviation of the hedonic evaluation in a 9-point scale; ^2^ Results expressed in percentage of consumer indicating the intention for each formulation. ^3^ Means followed by different letters present significative differences (*p* <0.05); ^4^ Adjusted residuals; absolute value >1.96.

## Supplementary Materials

The following are available online at https://www.mdpi.com/2304-8158/9/2/206/s1, Raw data for wine and garlic to replace nitrite, Patarata et al.
